# Sex Differences in BNST and Amygdala Activation by Contextual, Cued, and Unpredictable Threats

**DOI:** 10.1523/ENEURO.0233-21.2021

**Published:** 2022-01-05

**Authors:** Louise Urien, Elizabeth P. Bauer

**Affiliations:** Departments of Biology and Neuroscience and Behavior, Barnard College of Columbia University, New York, NY 10027

**Keywords:** amygdala, conditioning, fear, FOS, threat

## Abstract

Fear and anxiety can be described as emotional and physical responses to predictable and unpredictable threats. While the amygdala is necessary for context and cued fear conditioning, the bed nucleus of the stria terminalis (BNST) is important for anxiety-like behavior and conditioned responses to diffuse and/or unpredictable threats. However, we still lack knowledge about how the BNST and amygdala nuclei act in coordination. Moreover, the incidence of anxiety disorders and posttraumatic stress disorder (PTSD) is substantially higher in women than in men, but most studies of fear conditioning are conducted in male rodents. Here, we asked whether the BNST and the lateral, basal, and central nuclei of the amygdala are active during the expression of fear conditioning in male and female rats using FOS immunohistochemistry. We first show that the BNST is indeed involved in context fear expression in males, but not in females. The lateral amygdala was active in both sexes during context fear expression. We next trained animals using tone cues paired with an unconditioned stimulus (US), or tone cues which were unpaired with the US, and thus nonpredictive. Females displayed greater fear expression to these unpaired tones than males. FOS was upregulated in both the BNST and the basal amygdala during fear expression to unpaired tones in both sexes. The differential processing of fear responses by males and females highlights the need to acknowledge sex differences in conditioned fear memory.

## Significance Statement

The prevalence of anxiety disorders and posttraumatic stress disorder (PTSD) is substantially higher in women than in men. Abnormalities in conditioned fear characterize many of these disorders. Sex differences have been reported for some forms of fear conditioning, but the neural circuits underlying these differences are not fully understood. Here, we quantified neural activity in three regions of the amygdala as well as the bed nucleus of the stria terminalis (BNST) in response to fear expression to context and cued fear conditioning. Our results indicate different patterns of activity in the BNST and amygdala in males and females during context fear expression. Second, while females showed enhanced behavioral responding to unpaired tones, the BNST and basal nucleus of the amygdala were similarly activated in males and females.

## Introduction

Atypical fear learning, expression, and extinction characterize many stress-related psychopathologies (Gorman et al., 2000; Milad et al., 2006; Maren and Holmes, 2016). Incorrectly attributing danger to a safety cue is a hallmark of anxiety disorders and posttraumatic stress disorder (PTSD). Moreover, fear responses are often linked to a specific context in which the environment itself is threatening. For example, patients suffering from PTSD exhibit “re-experiencing” when they are reminded of a traumatic event by being exposed to the same environment previously associated with the trauma. Recent studies in both men and women reveal that these disorders are associated with changes in neural activity in both the amygdala and the bed nucleus of the stria terminalis (BNST; Sripada et al., 2012; Avery et al., 2016; Brinkmann et al., 2017a,b; Rabellino et al., 2018; Shackman and Fox, 2021). However, the prevalence of anxiety disorders and PTSD is substantially higher in women than in men (Altemus et al., 2014; Kimerling et al., 2018).

While decades of research have focused on the role of the amygdala in Pavlovian fear conditioning (Fanselow and LeDoux, 1999; Pape and Pare, 2010), the BNST is emerging as a crucial contributor to conditioned fear. Early research in rodents using lesions and temporary inactivation established that the BNST is necessary for the expression but not the acquisition of context fear conditioning (Walker et al., 2003; Sullivan et al., 2004; Duvarci, et al., 2009). This led to the idea that the BNST is crucial for conditioning to sustained cues (including context) but not discrete cues (Davis et al., 2010). More recent work in both rodents and humans suggests that the BNST also contributes to conditioning to unpredictable cues (Figel et al., 2019; Goode et al., 2019; Naaz et al., 2019).

The BNST of males and females have clear volumetric and neurochemical differences (del Abril et al., 1987; Allen and Gorski, 1990; Hines et al., 1992) and areas of the BNST involved in reproductive function show different patterns of connectivity in males and females (Polston et al., 2004). Many of these differences are acquired during the early stages of development and are attributable to different hormonal influences (Stefanova and Ovtscharoff, 2007). Sexual dimorphism in the BNST could in part explain some differences observed in contextual fear conditioning, where female rodents exhibit less freezing behaviors than males (Archer, 1975; Maren, et al., 1994; Fanselow and Dong, 2010; Colon et al., 2018; Colon and Poulos, 2020). However, others find stronger conditioning in females (Moore et al., 2010; Keiser et al., 2017), while still others find no differences in context fear expression (Dachtler et al., 2011; Urien et al., 2021).

The BNST is often referred to as the extended amygdala (Alheid and Heimer, 1988; Swanson and Petrovich, 1998; Fox et al., 2015). It receives glutamatergic input from the basolateral nucleus of the amygdala (Krettek and Price, 1978; Smith and Paré, 1994; Paré et al., 1995), and it is reciprocally connected with the central nucleus (CE) of the amygdala (Sun and Cassell, 1993; Shin et al., 2008). All of these amygdala nuclei play a key role in the acquisition of Pavlovian cued fear conditioning (Repa et al., 2001; Herry et al., 2008; Ciocchi et al., 2010). While some studies have found reduced cued fear conditioning in females (Pryce et al., 1999; Baran et al., 2010), most find no sex differences (Baker-Andresen et al., 2013; Fenton et al., 2014; Voulo and Parsons, 2017; Day et al., 2020). Interestingly, female rodents show more generalization of fear to novel and safe contexts and cues (Day et al., 2016; Keiser et al., 2017). They also show deficits in conditioned inhibition compared with males (Krueger and Sangha, 2021). In human studies, females discriminate less between fear and safety signals (Lonsdorf and Kalisch, 2011; Gamwell et al., 2015).

This study directly compared neural activation, as indexed by FOS immunohistochemistry, in four interconnected regions: the BNST, the LA, the CE, and the BA in both male and female rats after conditioned fear expression. Animals underwent context fear conditioning, or either paired or unpaired cued fear conditioning. By directly comparing behavior and patterns of neuronal activation, we aimed to reveal both similarities and differences in fear expression in males and females.

## Materials and Methods

### Subjects

Male (*n* = 36; 250–325 g) and female (*n* = 44; 225–275 g) Sprague Dawley rats were purchased from Charles River Laboratories. They were housed in pairs, with *ad libitum* access to food and water, and maintained on a 12/12 h light/dark cycle. All behavioral experiments were performed during the light cycle between 8 and 11 A.M. All procedures were approved by Columbia University’s Animal Care and Use Committee in accordance with the National Institutes of Health *Guide for the Care and Use of Laboratory Animals*.

### Context fear conditioning

On day 1, rats were habituated to the context for 10 min which consisted of a standard conditioning chamber with a metal grid floor (Coulbourn Instruments). On day 2, animals were placed in the conditioning chamber for 10 min and received three unsignaled footshocks (0.5 mA, 1 s). Animals in the “no shock” group were placed in conditioning chambers for 10 min on day 2 but did not receive shocks. On day 3, animals were returned to the conditioning chamber for 10 min. Total time spent freezing after the shock during conditioning (30 s) and to the context during testing (freezing time per minute over 10 min) was manually scored offline for each animal by an observer blind to group assignment.

### Cued fear conditioning

On day 1, rats were habituated to the context A for 10 min, which consisted of a standard conditioning chamber with a metal grid floor (Coulbourn Instruments). On day 2, animals were placed in the conditioning chamber in context A for 10 min and received three signaled footshocks (0.5 mA, 1 s) preceded by a tone stimulus (80 dB, 30 s). Animals in the no shock group were placed in the conditioning chamber in context A for 10 min, heard the tone but did not receive shocks. On day 3, animals were placed in context B and received five tones (80 dB, 30 s). Context B contained a black Plexiglas floor washed with peppermint soap, different wall materials (clear plastic or metal) and different light placement. Total time spent freezing to the tone was manually scored offline for each animal by an observer blind to group assignment.

### Unpaired cued fear conditioning

On day 1, rats were habituated to context A for 10 min as described above. On day 2, animals were placed in the conditioning chamber in context A for 10 min and received three footshocks [0.5 mA, 1 s, unconditioned stimulus (US)1: 329 s; US2: 417 s; US3: 486 s] and three 30-s tone stimuli [80 dB, 30 s; conditioned stimulus (CS)1: 250 s; CS2: 430 s; CS3: 520 s]. The tone CSs were explicitly unpaired with footshocks. On day 3, animals were placed in context B as described above and received five tones (80 dB, 30 s). Total time spent freezing to the tone was manually scored offline for each animal by an observer blind to group assignment.

### Immunohistochemistry

A total of 60–90 min after the completion of behavioral testing, animals were given an overdose of sodium pentobarbital (100 mg/kg) and perfused transcardially with 0.1 m PBS and 4% paraformaldehyde in 0.1 m PB. After perfusions, brains were postfixed for 24 h and transferred to a 20% sucrose solution for at least 48 h. Tissue was sectioned at 80 μm using a Vibratome. A total of 10–12 slices per animal was collected, covering the rostro-caudal extent of the BNST, and 20–24 slices were collected covering the rostro-caudal extent of the BLA and CE. Every other slice was processed and analyzed, so there was no need to correct for double counting. Tissue was washed first in 0.1 m PB three times, then in PB with 1% Triton X-100 (PBT) three times for 5 min each wash. Slices were blocked in 2% normal goat serum (Vector Laboratories) in PBT for 1 h. Slices were then incubated for 48 h at 4°C in primary polyclonal rabbit anti-cFOS antibody (1:1000, Abcam ab190289) in block solution. Slices were washed in PBT and incubated with secondary biotinylated goat anti-rabbit antibody (1:200; Vector Labs BA1000) for 1 h at room temperature. Slices were processed with avidin-biotin horseradish peroxidase complex (Vectastain Elite ABC kit, Vector Labs PK6100). Horseradish peroxidase was visualized with 3,3’diaminobenzidine (Sigma-Aldrich D5905) in a 3 m sodium acetate buffer containing 0.05% H_2_O_2._ and enhanced with nickel. Slices were washed in PBS, mounted on slides and coverslipped.

### Microscopy

For each brain analysis, we selected 80 μm slices covering the anterior-posterior axes of each structure (BNST: AP +0.12 to AP −0.36; CE: AP −2.50 to AP −3.0; BLA: AP −2.75 to AP −3.25). Delineation of the borders of each structure, and measurement of the area of each structure were made using NIS element software and ImageJ. We analyzed FOS expression in the dorsal anterolateral and anteromedial portions of the BNST as these areas are involved in context fear expression (Haufler et al., 2013). Three regions of the amygdala were analyzed: the CE, LA, and BA. Within the CE, we analyzed both the lateral (CeL) and medial (CeM) divisions. FOS-positive cells were counted manually using ImageJ software. For each animal, we analyzed left and right hemispheres separately to reveal potential lateralization of FOS activity. Two to three samples per hemisphere were averaged together and divided by the surface area of the structure to normalize cell counts by area. Thus, for each animal we obtained two measurements of FOS activity per brain structure: one measurement for the left hemisphere and one for the right hemisphere.

### Statistical analyses

All statistical analyses were performed using SPSS software. Two-way repeated-measures ANOVAs assessed the effect of sex and behavioral group on freezing behavior. When appropriate, we conducted one-way ANOVAs and Tukey’s HSD *post hoc* tests. To analyze FOS expression, mixed model ANOVAs were conducted with sex and behavioral group as between-subject factors, and laterality as a nested factor. When appropriate, we conducted one-way ANOVAs and Tukey’s HSD *post hoc* tests. We tested for correlations between FOS expression and freezing behavior using Spearman’s rank order correlation, as freezing behavior was not normally distributed. For all experiments, differences were considered significant when *p *<* *0.05.

## Results

### Context fear conditioning

**Figure 1.**
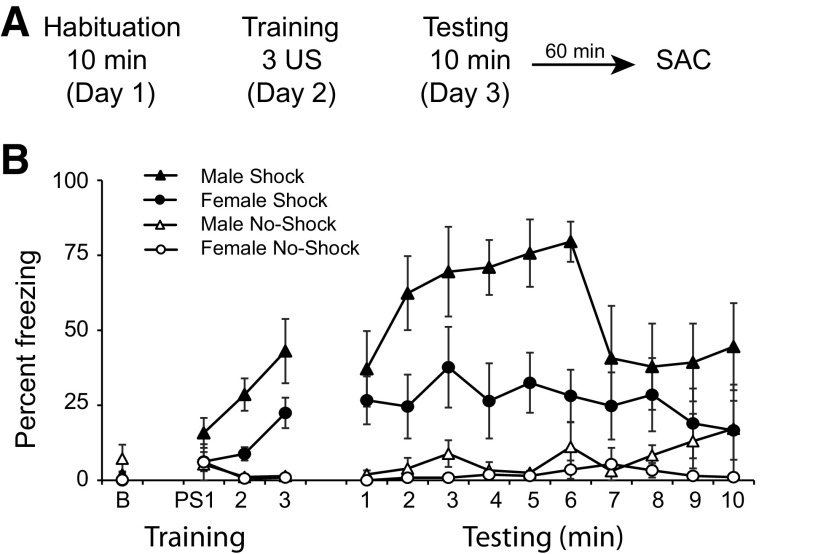
Context fear expression in males and females. ***A***, Schematic of behavioral protocol. ***B***, Percent freezing +/- s.e.m. during training at baseline (B) and after three unsignaled footshocks (PS1, PS2, PS3) or the equivalent time points in animals receiving no shocks. Percent freezing during the 10 min of testing in males and females.

Our first experiment measured upregulation of the immediate early gene FOS in the BNST and amygdala of males and females after context fear expression (Fig. 1). Animals that underwent behavioral testing were habituated to the training context for 10 min on day 1. During training on day 2, animals were placed in the conditioning chamber for 10 min and received three unsignaled footshocks (males *n* = 7; females *n* = 8) or no footshocks (males *n* = 6, females *n* = 8). Twenty-four hours later, animals were returned to the conditioning chambers for 10 min. One hour after the completion of testing, animals were perfused.

To confirm that there were no a priori differences between groups, we analyzed freezing in the 30 s before the first shock on training day using a two-way ANOVA. We found no differences in freezing behavior, with no main effect of behavioral group, sex or sex × group interaction (*p*s > 0.05). To assess acquisition of context fear conditioning, we analyzed freezing during the 30 s after each shock (postshock freezing) and during the corresponding time points in the no shock groups. A two-way repeated measures ANOVA revealed a significant interaction between time and behavior (*F*_(2,50)_ = 8.71; *p* < 0.001) with shock animals freezing more than no shock animals over time. There was no significant interaction between time and sex or time × sex × behavioral group interaction (*p*s > 0.05).

To assess context fear expression, we analyzed freezing during the 10 min test in males and females receiving shocks or no shocks using a two-way repeated measures ANOVA. We found a significant effect of behavior, with animals receiving shocks freezing more than no shock animals (*F*_(1,25)_ = 36.1; *p* < 0.0005), but no significant effect of time (*F*_(3.89, 97.21)_ = 1.947; *p* = 0.11 with a Greenhouse–Geisser correction). The interaction between behavioral group and sex showed a trend toward significance *F*_(1,25)_ = 3.88; *p* = 0.06; Fig. 1*B*).

**Figure 2.**
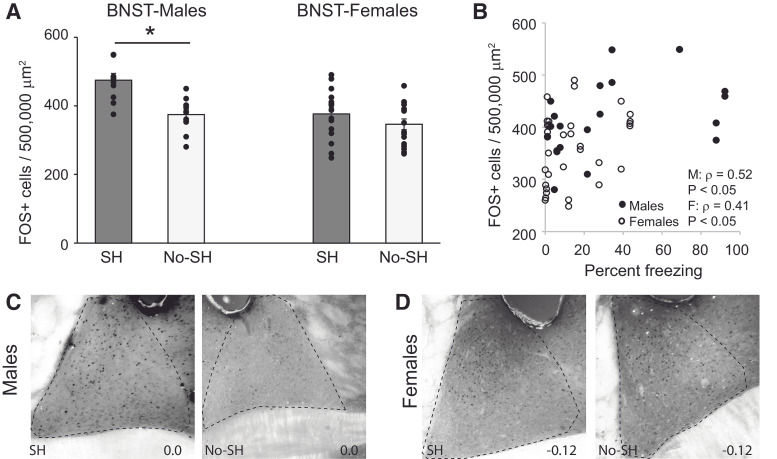
Differential effects of context fear expression on FOS immunoreactivity in the BNST in males and females. ***A***, Mean +/- s.e.m. FOS+ cells in the BNST in the shock (SH) no shock (No-SH) in males and females. Asterisk indicates a significant difference in FOS between males receiving shocks and males receiving no shocks, *p < 0.01. ***B***, Correlation between the number of FOS+ cells in the BNST and freezing behavior in males and females. Photomicrographs of the anterolateral BNST in males (***C***) and females (***D***) in the shock and no shock conditions. Anterior-posterior distance from bregma (in mm) is denoted in the lower right of each image.

The number of FOS-positive cells was quantified across the two behavioral groups in the BNST, CE, LA, and BA. As there have been reports of lateralized activity in both the BNST (Luyck et al., 2020) and amygdala (Baker and Kim, 2004) following fear conditioning, we used mixed model ANOVAs to analyze FOS activity. A nested design accounted for laterality (left vs right hemisphere) within subjects and multiple measures (sex and behavioral group) between subjects. For the BNST, a mixed model ANOVA revealed a significant interaction between sex and behavioral group (*F*_(1,23)_ = 7.44; *p* = 0.01; Fig. 2*A*), but no effect of laterality (*F*_(1,23)_ = 0.177; *p* = 0.68). We then analyzed males and females separately. For males, a one-way ANOVA revealed a main effect of behavior (*F*_(1,9)_ = 13.03, *p* < 0.01) with more FOS-positive cells in the shock group than the no shock group (Fig. 2*A*,*C*). In contrast, there were no significant differences in the number of FOS positive cells in the BNST between the two female behavioral groups (*F*_(1,14)_ = 0.85; *p* = 0.37; Fig. 2*A*,*D*). These data confirm that male but not female rats have higher levels of neuronal activity in the BNST during expression of contextual fear. We then examined whether freezing behavior correlated with FOS activity in the BNST. Because we found differences in FOS expression between sexes, we analyzed males and females separately. FOS expression was indeed correlated with freezing behavior in both males (ρ = 0.52, *p* < 0.05) and females (ρ = 0.41, *p* < 0.05; Fig. 2*B*).

**Figure 3.**
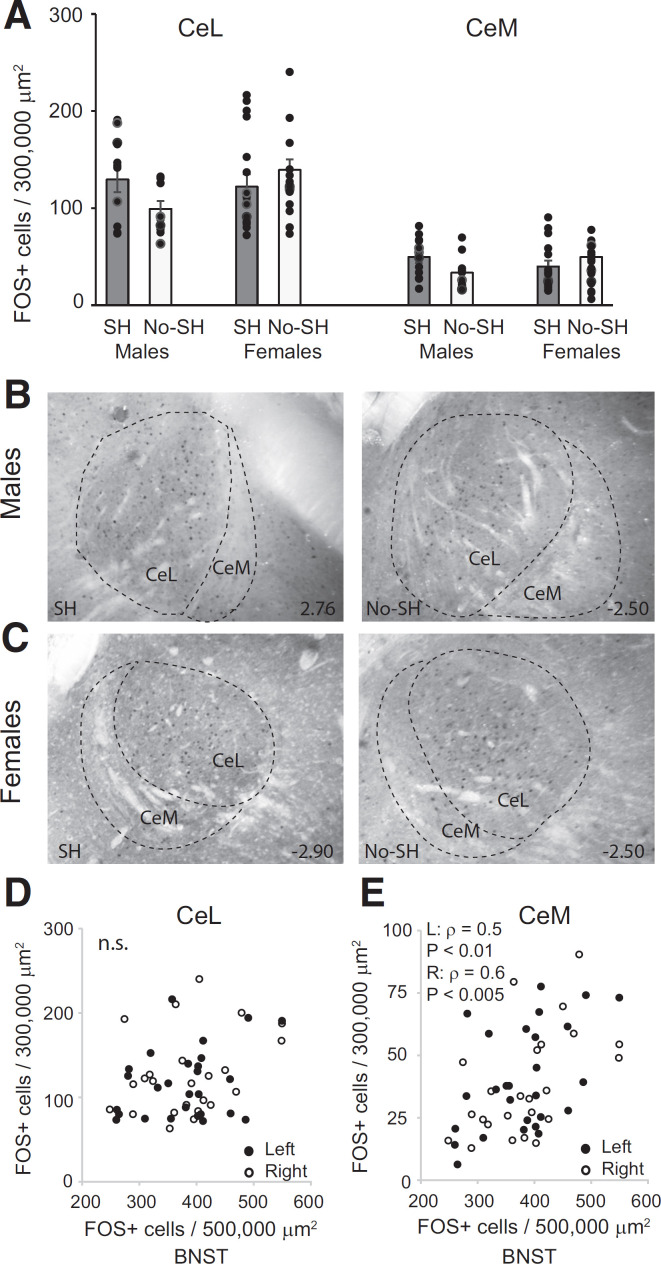
Context fear expression does not upregulate FOS in the CeL or CeM in males and females. ***A***, Mean +/- s.e.m. FOS+ cells in the CeL and CeM in the shock (SH) and no shock (No-SH) in males and females. Photomicrographs of the CE in males (***B***) and females (***C***) in the shock and no shock conditions, subdivided into CeL and CeM. Anterior-posterior distance from bregma (in mm) is denoted in the lower right of each image. ***D***, Correlation between the number of FOS+ cells in the CeL and BNST in all animals. ***E***, Correlation between the number of FOS+ cells in the CeM and BNST in all animals.

We analyzed FOS expression in the CeL and CeM separately. In the CeL, a mixed model ANOVA revealed no significant effect of behavioral group (*F*_(1,22)_ = 0.81; *p* = 0.38), sex (*F*_(1,22)_ = 0.57; *p* = 0.46) or interaction (*F*_(1,22)_ = 0.74; *p* = 0.4; Fig. 3*A–C*). While there was a significant effect of laterality (*F*_(1,22)_ = 4.96; *p* < 0.05), with greater FOS activation in the right CeL, there was no interaction between laterality and either behavior or sex (*p*s > 0.05). Further, there was so significant correlation between FOS expression and freezing (left hemisphere ρ = 0.06; *p* = 0.76; right hemisphere: ρ = 0.02; *p* = 0.94). In the CeM, a mixed-model ANOVA revealed no significant effect of behavioral group (*F*_(1,23)_ = 0.85; *p* = 0.37), sex (*F*_(1,23)_ = 0.04; *p* = 0.84), or interaction *F*_(1,23)_ = 1.23; *p* = 0.28, and no effect of laterality *F*_(1,23)_ = 0.55; *p* = 0.47; Fig. 3*A–C*). Further, there was so significant correlation between FOS expression and freezing (left hemisphere ρ = 0.08; *p* = 0.69; right hemisphere: ρ = 0.22; *p* = 0.29).

As the CeM receives direct input from the anterolateral BNST (Dong and Swanson, 2004), we correlated FOS expression between these two areas, analyzing left and right hemispheres separately. Indeed, FOS expression was correlated between the CeM and BNST (left hemisphere: ρ = 0.5; *p* < 0.01; right hemisphere: ρ = 0.6; *p* < 0.005), but not between the CeL and BNST (left hemisphere: ρ = 0.21; *p* = 0.3; right hemisphere: ρ = 0.28; *p* = 0.18; Fig. 3*D*,*E*).

**Figure 4.**
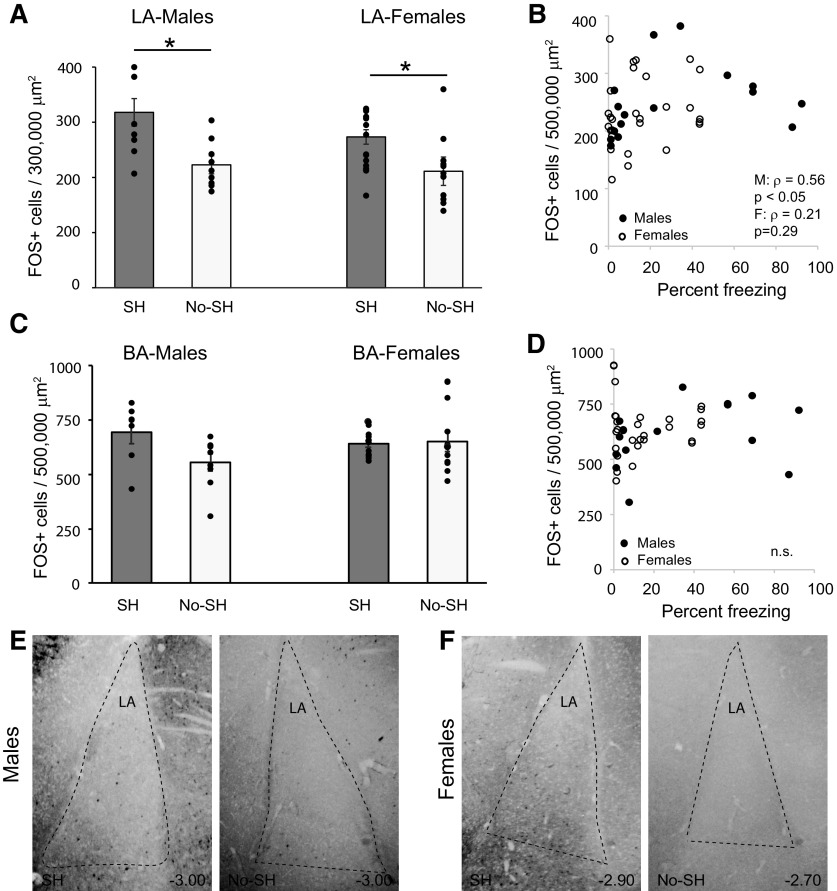
Context fear expression upregulates FOS in the LA but not BA in males and females. ***A***, Mean +/- s.e.m. FOS+ cells in the LA in the shock (SH) and no shock (No-SH) in males and females. Asterisk indicates a significant difference in FOS between shock animals and no-shock animals, *p = 0.01. ***B***, Correlation between the number of FOS+ cells in the LA and freezing behavior in males and females. ***C***, Mean +/- s.e.m. FOS+ cells in the BA in the shock (SH) and no shock (No-SH) groups in males and females. ***D***, Correlation between the number of FOS+ cells in the BA and freezing behavior in males and females. Photomicrographs of the LA in males (***E***) and females (***F***) in the shock and no shock conditions. Anterior-posterior distance from bregma (in mm) is denoted in the lower right of each image.

In the LA, a mixed-model ANOVA revealed a significant effect of behavior (*F*_(1,18)_ = 4.66; *p* < 0.05) with greater FOS activity in the shock animals. There was no main effect of sex (*F*_(1,18)_ = 0.28, *p* = 0.60) or interaction (*F*_(1,18)_ = 0.46, *p* = 0.51), and no effect of laterality (*F*_(1,18)_ = 0.84, *p* = 0.37; Fig. 4*A*,*E*,*F*). FOS expression was correlated with freezing behavior for males (ρ = 0.56; *p* < 0.05) but not females (ρ = 0.21; *p* = 0.29; Fig. 4*B*). Within the BA, we found no significant effect of behavioral group (*F*_(1,17)_ = 1.77, *p* = 0.2), sex (*F*_(1,17)_ = 0.58, *p* = 0.4) or interaction (*F*_(1,17)_ = 0.03, *p* = 0.86), and no effect of laterality (*F*_(1,17)_ = 0.08, *p* = 0.78; Fig. 4*C*). Correlating FOS expression with freezing behavior revealed a trend toward significance for males (ρ = 0.46; *p* = 0.06) but not females (ρ = −0.08; *p* = 0.7; Fig. 4*D*). In sum, context expression upregulated FOS expression in the LA and we found no differences in FOS expression between males and females in any of the three amygdalar regions.

### Paired and unpaired cued fear conditioning

**Figure 5.**
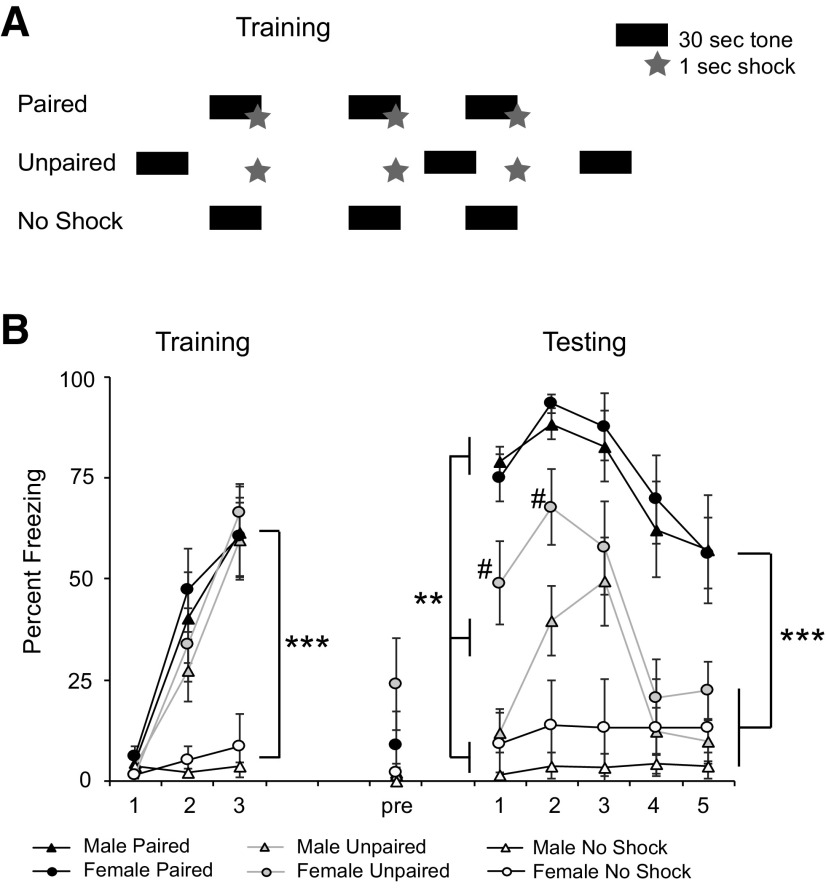
Conditioning in males and females to paired and unpaired cues. ***A***, Schematic of the training protocol in three behavioral groups: paired (P), unpaired (UP), and no shock. Bars represent 30-s CS tones. Stars represent 1-s footshocks. Animals in the cued group received three CS tones cotermininating with footshocks. Animals in the unpaired group received tones and shocks which were explicitly unpaired. Animals in the no-shock group received tones but no shocks. ***B***, Percent freezing +/- s.e.m. during each of the three tones during training, to the novel context before tone delivery during testing (pre) and during the five tone presentations during testing. Asterisks indicate differences in freezing between all three behavioral groups during the first three tones, and between cued animals and the other four groups during the last two tones; # indicates a difference in freezing between males and females in the unpaired group during the first two tones; **p < 0.005, ***p < 0.0001, #*p* < 0.05.

The BNST contributes to fear expression when cues are nonpredictive (Goode et al., 2019) while the LA is important for responses to cued fears (Repa et al., 2001). We trained animals with two different fear paradigms in which the cues either predicted a footshock (paired) or not (unpaired). During training, one group of animals was placed in the conditioning chamber A for 10 min and received three signaled footshocks with a 30-s tone co-terminating with the shock (males *n* = 6; females *n* = 10). In this behavioral design, the tone is paired with the shock and is thus a strong predictor of the shock. A second group of animals received the tones but no footshock (males *n* = 7; females *n* = 8; no shock). A third group of animals received three footshocks that were not paired with the tone (males *n* = 10; females *n* = 10; unpaired). The tone CS was thus unpaired with the shock and a poor predictor of the shock (Fig. 5*A*). Both males and females acquired freezing after being shocked (postshock freezing), in comparison to animals who just heard the tone (main effect of behavior *F*_(2,49)_ = 21.45; *p* < 0.0001; Fig. 5). There was no main effect of sex (*F*_(1,45)_ = 0.53; *p* = 0.5) or interaction between sex and behavioral group (*F*_(2,45)_ = 0.016; *p* = 0.98).

Twenty-four hours later, animals were placed in a different conditioning chamber and received five CS tones. One hour after the completion of testing, animals were perfused. We first analyzed pre-CS freezing during the 30 s before the first tone. A two-way ANOVA (sex × behavioral group) revealed no effect of behavioral group (*F*_(1,45)_ = 1.48; *p* = 0.24) sex (*F*_(1,45)_ = 3.323; *p* = 0.08) or interaction (*F*_(2,45)_ = 1.27; *p* = 0.29). As animals were trained in context A, and tested in context B, it is possible some context generalization occurred. We thus performed a one-sample *t* test on pre-CS freezing levels to determine whether freezing was significantly different from zero. Pre-CS freezing was not significantly different from zero for the animals receiving paired training (males: *p* = 0.36; females: *p* = 0.31) or unpaired training (males: *p* = 0.17; females *p* = 0.07). Finally, for animals receiving unpaired training, we tested whether freezing to the first tone was significantly different from pre-CS freezing. Unpaired *t* tests revealed that both males (*p* = 0.03) and females (*p* = 0.009) exhibited significantly more freezing to the tone than the pre-CS period (Fig. 5*B*). Together, these data suggest that there was no significant context generalization between training and testing contexts.

We next analyzed freezing behavior to the five tone presentations. A two-way repeated measures ANOVA revealed a significant interaction between tone and behavioral group (*F*_(8,180)_ = 4.5; *p* < 0.0001). We then performed two-way ANOVAs for each of the five tones with Tukey’s HSD *post hoc* tests to determine significant differences between behavioral groups. During the first three tones, there were significant differences between all three behavioral groups (paired, unpaired and no-shock; *p*s < 0.005). By tones 4 and 5, there were differences between the paired groups and the other two behavioral groups (*p*s < 0.0001) but no difference between animals using unpaired tones and the no shock groups (*p* = 0.71, *p* = 0.63). We also found a significant effect of sex on freezing behavior for tones 1 (*p* = 0.027) and 2 (*p* = 0.031, Tukey’s HSD), with females in the unpaired group freezing more than males trained in the unpaired group (*p* = 0.004, *p* = 0.04, Student’s *t* test; Fig. 5*B*). In sum, animals receiving paired training froze most, while animals trained using unpaired tone-shocks showed an intermediate amount of fear expression during early testing, but extinguished quickly. In addition, female rats showed greater fear expression in the unpaired group than males.

**Figure 6.**
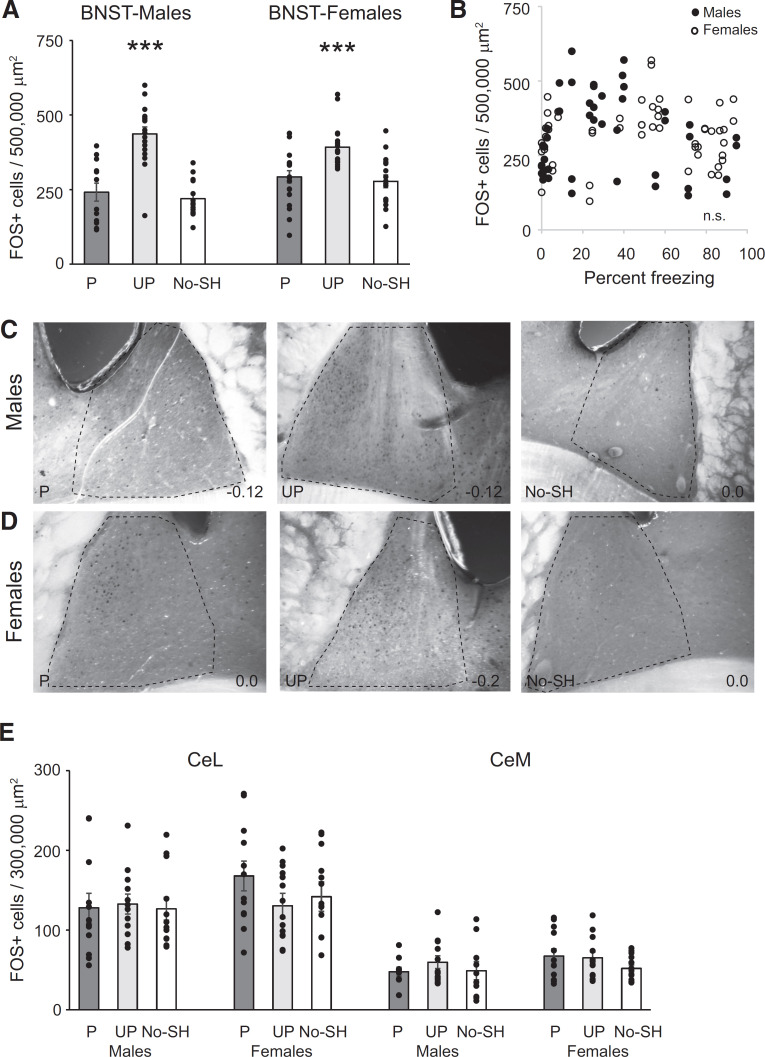
Conditioning to unpaired cues upregulates FOS in the BNST but not the CE in males and females. ***A***, Mean +/- s.e.m. FOS+ cells in the BNST in the paired (P), unpaired (UP), and no shock (No-SH) group in males and females. Asterisk indicates a significant difference in FOS between animals in the unpaired group and the other two behavioral groups, ***p < 0.001. ***B***, Correlation between the number of FOS+ cells in the BNST and freezing behavior in all males and females. Photomicrographs of the BNST in males (***C***) and females (***D***) in the P, UP, and No-SH groups. Anterior-posterior distance from bregma (in mm) is denoted in the lower right of each image. ***E***, Mean +/- s.e.m. FOS+ cells in the CeL and CeM in the paired (P), unpaired (UP), and no shock (No-SH) group in males and females.

We then analyzed the level of FOS activity in the BNST, CE, LA, and BA in the three groups of animals. A mixed-model ANOVA of FOS-positive cells in the BNST revealed a main effect of behavior (*F*_(2,43)_ = 21.37; *p* < 0.001), but no effect of sex (*F*_(1,43)_ = 0.85; *p* = 0.36) or interaction (*F*_(2,43)_ = 2.11; *p* = 0.13) and no effect of laterality (*F*_(1,43)_ = 0.07; *p* = 0.79; Fig. 6*A*,*C*,*D*). Tukey’s HSD *post hoc* test revealed a significant difference in FOS expression between animals trained with unpaired tones and the other two behavioral groups (*p*s < 0.001), but no upregulation of FOS in the BNST in the paired group compared with the no-shock group (*p* = 0.71). There was no correlation between FOS expression and freezing behavior (males: ρ = 0.04; *p* = 0.82; females: ρ = 0.17; *p* = 0.23; Fig. 6*B*). These data suggest that in males and females the BNST is involved in responding to cues which were unpaired with shocks during training and were thus nonpredictive.

We next analyzed FOS upregulation in the amygdala. Within the CeL, we found no significant effect of behavior (*p* = 0.93), sex (*p* = 0.11), interaction between sex × behavior (*p* = 0.83), and no effect of laterality (*p* = 0.89; Fig. 6*E*). There was no correlation between FOS activity in the CeL and freezing behavior (left hemisphere: *p* = 0.51; right hemisphere *p* = 0.88). While there was no significant correlation between activity in the left CeL and left BNST (*p* = 0.14) there was a significant correlation in the right hemisphere (ρ = 0.38; *p* = 0.02). Within the CeM, we found no significant effect of behavior (*p* = 0.31), sex (*p* = 0.16), interaction between sex × behavior (*p* = 0.76), and no effect of laterality (*p* = 0.92; Fig. 6*E*). There was no correlation between FOS activity in the CeM and freezing behavior (left hemisphere: *p* = 0.63; right hemisphere *p* = 0.77), or between FOS activity in the CeM and the BNST (left hemisphere: *p* = 0.1; right hemisphere *p* = 0.68).

**Figure 7.**
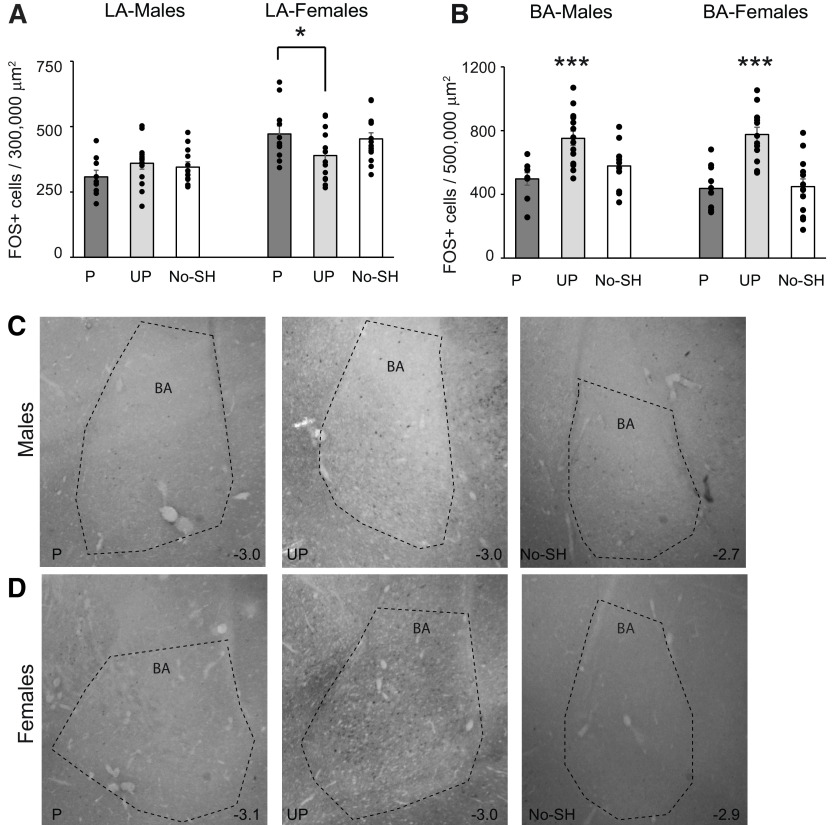
Conditioning to paired and unpaired cues upregulates FOS in the BLA. ***A***, Mean +/- s.e.m. FOS+ cells in the LA in the paired (P), unpaired (UP), and no shock (No-SH) group in males and females. Asterisk indicates a significant difference in FOS between females in the paired group and the unpaired group. ***B***, Mean +/- s.e.m. FOS+ cells in the BA in the paired (P), unpaired (UP), and no shock (No-SH) group in males and females. Asterisk indicates significant higher numbers of FOS-positive cells in the unpaired group than in the two other groups in males and females (*p*s < 0.005). ***C***, ***D***, Photomicrographs of the BA in males (***C***) and females (***D***) in the cued, random and no shock groups. Anterior-posterior distance from bregma (in mm) is denoted in the lower right of each image.

Within the LA, we found a main effect of sex with more FOS-positive cells overall in females than in males (*F*_(1,29)_ = 31.56; *p* < 0.001), and a sex × behavior interaction (*F*_(2,29)_ = 4.81; *p* < 0.05), but no effect of laterality (*F*_(1,29)_ = 0.54; *p* = 0.47; Fig. 7*A*,*C*,*D*). We then analyzed males and females separately. In females, one-way ANOVA revealed a significant effect of behavioral group (*F*_(2,16)_ = 4.03; *p* = 0.03). Tukey’s *post hoc* tests revealed a difference in FOS expression between the paired and unpaired groups (*p* = 0.03), but not between other groups. In males, a one-way ANOVA revealed no significant effect of behavioral group (*F*_(2,13)_ = 1.21; *p* = 0.33). Because we found that behavior affected FOS expression in females, we analyzed whether FOS activity correlated with freezing in females. There was a trend toward a significant correlation within the left hemisphere (ρ = 0.44; *p* = 0.053) but not the right (ρ = 0.08; *p* = 0.75). In males, there was no correlation between FOS expression and freezing behavior: left hemisphere: ρ = −0.12; *p* = 0.63 and right hemisphere: ρ = 0.16; *p* = 0.53.

In the BA, a mixed-model ANOVA revealed a main effect of behavioral group (*F*_(2,30)_ = 8.41; *p* < 0.001), but no effect of sex (*F*_(1,30)_ = 0.25; *p* = 0.62) or interaction (*F*_(2,30)_ = 0.77; *p* = 0.47) and no effect of laterality (*F*_(1,30)_ = 0.23; *p* = 0.63) Tukey’s HSD *post hoc* tests revealed higher numbers of FOS-positive cells in the unpaired group than in the other two groups (*p*s < 0.005; Fig. 7*B–D*). There was no significant correlation between FOS activity in the BA and freezing behavior (left hemisphere: ρ = 0.13; *p* = 0.44; right hemisphere: ρ = −0.05; *p* = 0.8). In sum, for females, we observed opposite responses in the BLA, with the BA being more active during fear expression to unpaired cues, and the LA being more active during paired cued fear expression. In males, the BA was most active during fear expression to unpaired cues.

## Discussion

In this study, we used context and cued fear conditioning paradigms combined with immunohistochemistry to compare neuronal activity in the BNST and amygdala of males and females rats and examine possible sex differences. In males, we found that the BNST and LA were active during context fear expression. In females, only the LA was active during context fear expression. In response to tones which were unpaired with shocks during training, females exhibited a greater freezing response than males, but both sexes showed increased activity in the BNST and BA.

While sex differences in context fear conditioning have been previously reported, some have found stronger conditioning in males (Maren et al., 1994; Anagnostaras et al., 1998; Colon et al., 2018), stronger conditioning in females (Moore et al., 2010; Keiser et al., 2017), or no differences (Kosten et al., 2006; Dachtler et al., 2011; Urien et al., 2021). Here, context fear expression upregulated FOS activity in the BNST in males, but not in females exposed to the same training paradigm. However, it is possible that a region of the BNST outside of the anterodorsal area analyzed in this study contributes to context fear expression in females. These data confirm the involvement of the BNST in context fear conditioning in males (Sullivan et al., 2004; Duvarci et al., 2009; Urien et al., 2021).

Context fear expression also resulted in upregulation of FOS in the LA in both sexes. Upregulation of ARC, FOS, and EGR1 in the LA in response to context fear conditioning has been reported (Malkani and Rosen, 2000; Wilson and Murphy, 2009; Chaaya et al., 2019) and the LA also exhibits context-dependent neuronal activity (Hobin et al., 2003). However, these previous studies were performed only in males. Our data suggests that the LA is similarly involved in context fear expression in females. Context fear conditioning requires animals to learn a conjunctive representation of context from multimodal sensory cues (Rudy and O’Reilly, 1999). One hypothesis for sex differences in context fear conditioning is that females are less efficient in forming context representations or use different strategies than males (Wiltgen et al., 2001; Monfort et al., 2015; Yagi et al., 2016). Our finding that FOS activation in the LA was increased in females during context fear expression supports this hypothesis, as FOS expression also increased in the LA following cued fear expression in females.

We performed correlation analyses for individual animals to assess whether freezing behavior was correlated with FOS activity in the BNST and amygdalar regions. Expression of context fear conditioning was indeed correlated with FOS activity in the BNST and LA, but not the CE or the BA. This analysis further suggests the involvement of the BNST and LA in context fear expression.

During our paired conditioning paradigm, males and females exhibited the same levels of freezing in response to a discrete cue. Indeed, there are few reports of sex differences in cued fear conditioning (Baker-Andresen et al., 2013; Fenton et al., 2014; Voulo and Parsons, 2017; Day and Stevenson, 2020), although some have reported reduced (Pryce et al., 1999; Baran et al., 2010) or enhanced (Gresack et al., 2009) freezing in females. Additionally, freezing to paired cues was higher than to unpaired cues in both sexes in agreement with previous studies (Goode et al., 2019; Glover et al., 2020) Interestingly, females responded more than males to the unpaired cues during fear expression. Knowing that females exhibit more generalization of fear (Day et al., 2016; Keiser et al., 2017; Krueger and Sangha, 2021), it is possible that the unpaired cues used in this conditioning paradigm created fear generalization in females.

However, there was no fear generalization to the testing context in animals trained with paired or unpaired cues. Specifically, there was no significant amount of freezing during the pre-CS period for any behavioral group. Thus, freezing to the tones during testing did not represent reactivation of a generalized context.

The earliest models of Pavlovian fear conditioning stated that learning depends on contingency, or the ability of the CS to predict US occurrence (Rescorla, 1968). Here, in the unpaired condition, we delivered CS cues which were explicitly unpaired with the US. Others have used the same behavioral paradigm of negatively correlating a CS and US which can result in the CS signifying a period of safety (Nahmoud et al., 2021). This is accompanied by depression of CS-induced activity in the LA (Rogan et al., 2005). Any weak learning of the association between the unpaired CS and US involves activation of the BNST (Goode et al., 2020). To our knowledge, the BNST’s involvement in fear expression to unpaired, nonpredictive cues has been assessed only in males. Here, we found upregulation of FOS in the BNST in both males and females.

FOS was also upregulated in the BA in response to unpaired cues in both males and females. Interestingly, the protein kinase ERK1/2 is also active in the BA after an unpaired conditioning protocol (Trifilieff et al., 2007). Individual neurons within the BA respond to safety cues presented alone, as well as safety and fear cues presented together (Sangha et al., 2013; Kyriazi et al., 2018, 2020). In contrast, neural activity in the LA seems to be downregulated in response to safety cues (Rogan et al., 2005). Moreover, the postsynaptic density on dendrites in the LA decreases after conditioning with unpaired CSs (Ostroff et al., 2010). Accordingly, we did not observe changes in FOS expression in the LA in the unpaired cue groups.

We performed correlation analyses in individual animals to assess whether freezing behavior in the paired, unpaired and no shock groups was correlated with FOS activity in the BNST and amygdalar regions. While there were no significant correlations in any regions, this is not surprising as freezing was highest in the paired groups, but FOS activity was highest in the unpaired groups in the BNST and BA.

Across our experiments, we did not observe any changes in FOS expression in the CE. Neuronal activity by somatostatin-containing neurons in the CeL contributes to fear acquisition, whereas conditioned fear responses are driven by output neurons in the CeM (Ciocchi et al., 2010; Sun et al., 2020). Interestingly, in animals undergoing context fear conditioning, we found that FOS expression was correlated in the BNST and CeM. Indeed, the anterolateral BNST projects more strongly to the CeM than the CeL (Dong and Swanson, 2004). Our data suggests that this BNST-CeM pathway is active during the expression of context fear conditioning. Future studies should analyze the contributions of different CE neuronal subtypes to fear conditioning, and compare males and females. Interestingly, BNST and CeM activity was not correlated in animals undergoing paired or unpaired conditioning, and BNST activity was not correlated with freezing behavior in these animals. Thus, while the BNST is recruited by both context fear expression and fear expression to unpaired stimuli, these contrasting results suggest possible circuit-level differences in BNST involvement in these two types of learning.

Surprisingly, expression of paired cued fear conditioning upregulated FOS in the LA in females but not in males. Many previous studies have demonstrated that the LA is required for the acquisition of cued fear conditioning and that acquisition upregulates the expression of immediate-early genes in the LA (Collins and Paré, 2000; Repa et al., 2001; Ploski et al., 2008). However, there are few studies measuring FOS in the LA following the expression of cued fear conditioning. Some find no increase in FOS in the LA (Hall et al., 2001), others find an increase in the most dorsal portion of the LA, but not in the ventral LA (Knapska and Maren, 2009); however, these experiments were performed only in males. There is evidence that despite similar behavioral responses to cued fear conditioning, males and females might possess fundamental sex differences in fear circuitry structure (Gruene et al., 2015). Thus, the possible differential contribution of the LA to fear expression in males and females should be explored further.

Overall, our findings suggest that in males, the BNST is important for processing fear responses to context and to unpredictable threats. In females, the LA is active during context fear expression. While both the BNST and the BA showed upregulation of FOS in response to cues unpaired to aversive shocks, females exhibited a stronger fear response to these cues. The aim of these experiments was to not only document differences in the magnitude of freezing behavior in males and females, but to consider the neural networks engaged by fear expression. Understanding which circuits are recruited during memory processes in both males and females will also help identify the mechanisms underlying the differential vulnerability of men and women to anxiety disorders and PTSD (McLean et al., 2011; Tronson and Keiser, 2019).

## Synthesis

Reviewing Editor: Christina Zelano, Northwestern University - Chicago Decisions are customarily a result of the Reviewing Editor and the peer reviewers coming together and discussing their recommendations until a consensus is reached. When revisions are invited, a fact-based synthesis statement explaining their decision and outlining what is needed to prepare a revision will be listed below. The following reviewer(s) agreed to reveal their identity: Joanna Dabrowska, Linnaea Ostroff.

Both reviewers agreed that the topic of this study is interesting and important. Both reviewers raised some important constructive questions and comments. Most importantly, details about the methods and experimental design were found to be unclear as presented in the manuscript, making it difficult to assess the validity of the study. These details will need to be clarified, and depending on these details, additional experiments may be required before the study is suitable for publication. Most importantly, whether the data collection and analysis were rigorously controlled needs to be made clear (see detailed questions in reviewer 1’s comments below, all of which need to be addressed). Both reviewers were also confused by the “random cued fear conditioning” group. Specifically, it isn’t clear what this training is either intended to do or actually doing. Actual random training would require some overlap between the tone and shock, but in a single session it would be difficult to avoid some partial reinforcement. It’s possible that the intervals between CS1/US1 and CS2/US3 could produce trace conditioning, but regardless, there is context conditioning. The freezing in context B could be context generalization, but we don’t know because the pre-CS freezing isn’t shown. If this group has sustained freezing then the data represent reactivation of a generalized context, but if CS freezing is lower than pre-CS there’s also reactivation of an inhibitory cue. Context B also adds novelty as a factor. Clarity in defining what is being compared and showing that the comparison is valid is needed. Showing and explaining the context freezing in the random group should explain what’s being compared. Both reviewers also raised questions regarding tissue collection, specific nuclei of the amygdala analyzed and many details regarding cFos quantification that are missing in the manuscript.

Reviewer’s original comments:

Reviewer 1:

Growing evidence suggests the crucial role of the BNST in mediating fear responses to unpredictable threats but the BNST role in females in mediating fear responses is still poorly understood. In the manuscript entitled “Sex differences in BNST and amygdala activation by contextual, cued and unpredictable threats” the authors investigate specific recruitment (by measuring cFos) of the BNST, the central, lateral, and basal amygdala to fear expression following fear conditioning to predictable, unpredictable, and contextual threats in both male and female rats. The authors attempt to tackle important questions, as higher fear reactivity to unpredictable threats is one of the characteristics of PTSD in humans and human females are at higher risk for anxiety and PTSD.

The experiments are well design, include rigorous controls and the results are convincing.

In random cued fear conditioning (which is no longer a ‘cued’ fear conditioning to be exact) the rats were placed in context B for the expression of conditioned fear. I do understand the rationale for context B as the authors were likely trying to capture activation in response to fear expression to random cues rather than the original context. However, since during random conditioning the cues did not become reliable predictors of shocks, the context very likely became one (according to competitive models of associative learning, Marlin 1981). Therefore, it would be interesting to have another control for the random cues, for which the randomly ‘cued’ conditioned rats are returned to the same context A. Then, a potential difference between rats subjected to random fear conditioning tested in context A (with cues) vs. tested in context B (with cues) can be extracted as a direct proxy for activation to unpredictable cues.

The authors measured fear expression in all groups as well as assessed cellular activation in the amygdala (lateral, basal) and the extended amygdala sub regions. Why correlation analyses were not performed for individual rats between fear expression and cFos? This might provide additional interesting information regarding recruitment of specific amygdala sub regions to various form of predictive vs. un-predictive fear expression.

Bregma levels (range) for each region in which the cFos analysis was performed should be provided. Specific divisions of the BNST and central amygdala (medial or lateral) should be described. This is important as, for example, cFos in the lateral amygdala (where CeA interneurons are located) might ask for a different interpretation than medial CeA where CeA output neurons are located. This should be also integrated in the discussion - as the BNST and CeA are mostly GABA-ergic structures, activation of BNST or CeA interneurons detected as cFos expression would not mean necessarily BNST or CeA activation but rather the opposite.

"Two to three samples per hemisphere were counted and averaged together” - please clarify the sentence.

Controls are appropriate but what was the rationale for including home cage control as these rats conceivably receive other sensory and social information than their experimental counterparts?

Related - the cFos activation (e.g. in the BNST) in no-shock and home cage controls seem rather high in females, which might explain why conditioning does not have any further effects. Were the animals handled before the experiments?

Please simplify titles of the results sections: for example ‘Upregulation of FOS in the amygdala and BNST depends on the predictability of the CS’ is misleading as in fact it rather depends on unpredictability of the CS.

Methods. Subjects - the total number of male and female rats should be provided. Software used for statistical analysis should be provided.

Please modify the title of Figure 5: Doubling the context fear training protocol in females increases FOS expression in the BNST and amygdala during context fear expression as ‘Doubling the context fear training’ is not self-explanatory.

Some references are missing:

---. 2018b. “Sexual Differentiation of Contextual Fear Responses.” Learning and Memory. - the authors are not provided

Some references need additional formatting, for example:

(Leonie Brinkmann et al. 2017; L Brinkmann et al. 2021;

(Lee and Davis 1997 is not correctly cited - as it’s not about contextual fear bur rather CRF-potentiated startle

Many references provided have only early in press versions without volume even though we published some time ago.

Reviewer 2:

This study compares fear conditioning memory expression and associated c-fos expression in the BNST and amygdala between female and male rats. The authors report two sex differences: females required more shocks in context conditioning than males to increase c-fos in the BNST, and females, but not males, had increased c-fos in lateral amygdala after retrieval of a fear cue. Much is unknown about whether the neural mechanisms of fear conditioning studied in males are similar in females, and it is important to conduct these experiments. However, as described in the manuscript some of the experimental design does not sound rigorous or even valid. If the experiments were done correctly this should be explained clearly, otherwise additional experiments or analysis need to be done.

Specific items that should be addressed:

1. Were the animals tested in the light or dark cycle?

2. Raw data should be displayed in all of the bar graphs.

3. A plot of freezing versus c-fos density would be useful as well.

3. Why is there a difference in c-fos in central amygdala between the home cage and box-only groups?

4. The 6-shock behavior data are not shown, and the design of the 3 versus 6-shock experiment is confusing. As described, it sounds like a new group of 5 female rats were trained post hoc and compared directly to the earlier groups of animals. This would of course be inappropriate- controls must be run in parallel.

5. The c-fos quantification is not described adequately and it is impossible to tell how rigorously controlled the analysis was. The authors state that “two to three samples per hemisphere were counted and averaged together.” Two to three samples (which I assume means sections) would be a 50% difference between animals in the area sampled, and would mean that laterality was not counterbalanced or accounted for and that some animals had different regions analyzed. The numbers in the graphs are not normalized, and there is no indication of the area of tissue that was quantified or the variability in sampling area between animals. The example images do not appear to show the same AP level. An explanation of how the authors ensured that all animals had exactly the same regions and area sampled, and exactly where (AP/DV coordinates of the sample frame) should be included.

6. The “random” tone-shock design is explicitly unpaired. The authors state that the “tone CS was designed to be a poor predictor of the shock,” but it is actually a negative predictor. The authors switch around the terms random, non-predictive, unpaired, unpredictable, and safety cue interchangeably, but these are not synonyms.

7. The activation of c-fos in basal amygdala in the unpaired groups is unsurprising, given that these groups experienced context fear conditioning and showed some generalization during testing. The surprising effect is the lack of c-fos activation in the LA of the males, and an explanation for this result should be offered.

## Author Response

Synthesis Statement for Author (Required):

Both reviewers agreed that the topic of this study is interesting and important. Both reviewers raised some important constructive questions and comments. Most importantly, details about the methods and experimental design were found to be unclear as presented in the manuscript, making it difficult to assess the validity of the study. These details will need to be clarified, and depending on these details, additional experiments may be required before the study is suitable for publication. Most importantly, whether the data collection and analysis were rigorously controlled needs to be made clear (see detailed questions in reviewer 1’s comments below, all of which need to be addressed). Both reviewers were also confused by the “random cued fear conditioning” group. Specifically, it isn’t clear what this training is either intended to do or actually doing. Actual random training would require some overlap between the tone and shock, but in a single session it would be difficult to avoid some partial reinforcement. It’s possible that the intervals between CS1/US1 and CS2/US3 could produce trace conditioning, but regardless, there is context conditioning. The freezing in context B could be context generalization, but we don’t know because the pre-CS freezing isn’t shown. If this group has sustained freezing then the data represent reactivation of a generalized context, but if CS freezing is lower than pre-CS there’s also reactivation of an inhibitory cue. Context B also adds novelty as a factor. Clarity in defining what is being compared and showing that the comparison is valid is needed. Showing and explaining the context freezing in the random group should explain what’s being compared. Both reviewers also raised questions regarding tissue collection, specific nuclei of the amygdala analyzed and many details regarding cFos quantification that are missing in the manuscript.

Reviewer’s original comments:

Reviewer 1:

Growing evidence suggests the crucial role of the BNST in mediating fear responses to unpredictable threats but the BNST role in females in mediating fear responses is still poorly understood. In the manuscript entitled “Sex differences in BNST and amygdala activation by contextual, cued and unpredictable threats” the authors investigate specific recruitment (by measuring cFos) of the BNST, the central, lateral, and basal amygdala to fear expression following fear conditioning to predictable, unpredictable, and contextual threats in both male and female rats. The authors attempt to tackle important questions, as higher fear reactivity to unpredictable threats is one of the characteristics of PTSD in humans and human females are at higher risk for anxiety and PTSD.

The experiments are well design, include rigorous controls and the results are convincing.

In random cued fear conditioning (which is no longer a ‘cued’ fear conditioning to be exact) the rats were placed in context B for the expression of conditioned fear. I do understand the rationale for context B as the authors were likely trying to capture activation in response to fear expression to random cues rather than the original context. However, since during random conditioning the cues did not become reliable predictors of shocks, the context very likely became one (according to competitive models of associative learning, Marlin 1981). Therefore, it would be interesting to have another control for the random cues, for which the randomly ‘cued’ conditioned rats are returned to the same context A. Then, a potential difference between rats subjected to random fear conditioning tested in context A (with cues) vs. tested in context B (with cues) can be extracted as a direct proxy for activation to unpredictable cues.

Response: Yes, our rationale for testing in context B was to separate freezing to the context from freezing to unpaired cues. Indeed, since the BNST is involved in context fear expression, testing in context A would upregulate FOS expression in this area, regardless of whether the BNST was involved in fear expression to unpaired cues. Further, it clarified the contribution of the LA and BA to context and cued fear conditioning. We agree with the reviewer that unpaired tone-shock pairings can produce context fear conditioning, and that context generalization could occur between the training and testing context. This could, theoretically, complicate the interpretation of our results. We therefore analyzed freezing in the 30 seconds prior to the first tone delivery during testing (pre-CS period; see Figure 5). From the Results section:

"We first analyzed pre-CS freezing during the 30 seconds prior to the first tone. A two-way ANOVA (sex X behavioral group) revealed no effect of behavioral group (F(1,45)=1.48; p=0.24) sex (F(1,45) = 3.323; p=0.08) or interaction (F(2,45) = 1.27; p=0.29). As animals were trained in context A, and tested in context B, it is possible some context generalization occurred. We thus performed a one-sample t-test on pre-CS freezing levels to determine whether freezing was significantly different from zero. Pre-CS freezing was not significantly different from zero for the animals receiving paired training (males: p=0.36; females: p=0.31) or unpaired training (males: p=0.17; females p=0.07). Finally, for animals receiving unpaired training, we tested whether freezing to the first tone was significantly different from pre-CS freezing. Unpaired t-tests revealed that both males (p=0.03) and females (p=0.009) exhibited significantly more freezing to the tone than the pre-CS period. Together, these data suggest that there was no significant context generalization between training and testing contexts.”

This analysis suggests that freezing to the tones during testing represents a behavioral response to the aversive tone and not to the combination of a new context, aversive through context generalization, plus the aversive tone. We have integrated this analysis into the Discussion.

Reviewer: The authors measured fear expression in all groups as well as assessed cellular activation in the amygdala (lateral, basal) and the extended amygdala sub regions. Why correlation analyses were not performed for individual rats between fear expression and cFos? This might provide additional interesting information regarding recruitment of specific amygdala sub regions to various form of predictive vs. un-predictive fear expression.

Response: We thank the reviewer for this suggestion and have included correlation analyses for all data (see Figures 2-4, 6, 7, and accompanying text in the Results). Expression of context fear conditioning (freezing) was indeed correlated with FOS activity in the BNST and LA, but not the CE and the BA. This complements our analysis showing that FOS activity is upregulated in the BNST and LA during context fear expression. We performed correlation analyses between freezing and FOS expression in animals receiving paired, unpaired or no-shock conditioning paradigms. We did not find significant correlations between FOS activity and freezing behavior in any of the structures. However, this is not surprising given that freezing was highest in the paired groups, but FOS activity was highest in the BNST and BA in the unpaired groups. We used the non-parametric Spearman’s correlation analysis for each, as freezing behavior was not normally distributed. This is explained in the Methods (Statistical Analyses).

Reviewer: Bregma levels (range) for each region in which the cFos analysis was performed should be provided. Specific divisions of the BNST and central amygdala (medial or lateral) should be described. This is important as, for example, cFos in the lateral amygdala (where CeA interneurons are located) might ask for a different interpretation than medial CeA where CeA output neurons are located. This should be also integrated in the discussion - as the BNST and CeA are mostly GABA-ergic structures, activation of BNST or CeA interneurons detected as cFos expression would not mean necessarily BNST or CeA activation but rather the opposite.

Response: Details on the range of Bregma levels are now provided in the Methods (Microscopy):

For each brain analysis, we selected 80 micron slices covering the anterior-posterior axes of each structure (BNST: AP +0.12 to AP -0.36; CE: AP -2.50 to AP -3.0; BLA: AP -2.75 to AP -3.25). Delineation of the borders of each structure, and measurement of the area of each structure were made using NIS element software and ImageJ.

Additionally, we have now provided Bregma levels in the lower right corner of each photomicrograph in each Figure.

We analyzed FOS expression in the dorsal anterolateral and anteromedial portions of the BNST, as these areas are involved in context fear expression (Haufler et al., 2013). This is now stated in the Methods. However, we were unable to reliably distinguish the boundary between the lateral and medial CE. Given the fact that the CEL contains both excitatory and GABAergic neurons, and contains two populations of neurons whose activity produces opposing effects on behavior (SOM+ and CRF+ neurons), a more thorough immunohistochemical examination of the contribution of these cell types to fear expression is an important future study. We have integrated these ideas into the Discussion.

Reviewer: “Two to three samples per hemisphere were counted and averaged together” - please clarify the sentence.

Response: We now include a more detailed explanation of our methodology (Methods: Microscopy):

"For each animal, we analyzed left and right hemispheres separately to reveal potential lateralization of FOS activity. Two to three samples per hemisphere were averaged together and divided by the surface area of the structure to normalize cell counts by area. Thus for each animal we obtained two measurements of FOS activity per brain structure: one measurement for the left hemisphere and one for the right hemisphere.”

Reviewer: Controls are appropriate but what was the rationale for including home cage control as these rats conceivably receive other sensory and social information than their experimental counterparts?

Response: Our rationale for including home cage animals was to obtain a measurement of baseline FOS activity. However, we agree with the reviewer that these are not the optimal controls, as they received different sensory input, and were not handled as frequently as the other behavioral groups. We have removed these animals from the manuscript and re-analyzed our data. Figures 2-4 (and accompanying text) reflect these changes.

Reviewer: Related - the cFos activation (e.g. in the BNST) in no-shock and home cage controls seem rather high in females, which might explain why conditioning does not have any further effects. Were the animals handled before the experiments?

Response: Yes, we agree with the reviewer that home-cage animals are not appropriate controls, and have removed these animals from the manuscript.

Reviewer: Please simplify titles of the results sections: for example ‘Upregulation of FOS in the amygdala and BNST depends on the predictability of the CS’ is misleading as in fact it rather depends on unpredictability of the CS.

Response: The subsections of the Results are now:

Context fear conditioning

Paired and unpaired cued fear conditioning

Reviewer: Methods. Subjects - the total number of male and female rats should be provided. Software used for statistical analysis should be provided.

Response: The total number of animals is now included in the first sentence of the Methods. SPSS software was used for statistical analysis. This is included in the last paragraph of the Methods.

Reviewer: Please modify the title of Figure 5: Doubling the context fear training protocol in females increases FOS expression in the BNST and amygdala during context fear expression as ‘Doubling the context fear training’ is not self-explanatory.

Response: Figure 5 has been removed (see response to Reviewer 2 below), and the figures renumbered.

Reviewer: Some references are missing:

---. 2018b. “Sexual Differentiation of Contextual Fear Responses.” Learning and Memory. - the authors are not provided

Some references need additional formatting, for example:

(Leonie Brinkmann et al. 2017; L Brinkmann et al. 2021;

(Lee and Davis 1997 is not correctly cited - as it’s not about contextual fear bur rather CRF-potentiated startle

Many references provided have only early in press versions without volume even though we published some time ago.

Response: We have corrected the reference section.

Reviewer 2:

This study compares fear conditioning memory expression and associated c-fos expression in the BNST and amygdala between female and male rats. The authors report two sex differences: females required more shocks in context conditioning than males to increase c-fos in the BNST, and females, but not males, had increased c-fos in lateral amygdala after retrieval of a fear cue. Much is unknown about whether the neural mechanisms of fear conditioning studied in males are similar in females, and it is important to conduct these experiments. However, as described in the manuscript some of the experimental design does not sound rigorous or even valid. If the experiments were done correctly this should be explained clearly, otherwise additional experiments or analysis need to be done.

Specific items that should be addressed:

1. Were the animals tested in the light or dark cycle?

Response: The animals were tested in the light cycle. This is now stated in the third sentence of the Methods.

2. Raw data should be displayed in all of the bar graphs.

Response: Raw data is now displayed in all bar graphs.

3. A plot of freezing versus c-fos density would be useful as well.

Response: We have now included correlation analyses for all data (see Figures 2-4, 6, 7, and accompanying text in the Results). Expression of context fear conditioning (freezing) was indeed correlated with FOS activity in the BNST and LA, but not the CE and the BA. This complements our analysis showing that FOS activity is upregulated in the BNST and LA during context fear expression. We performed correlation analyses between freezing and FOS expression in animals receiving paired, unpaired or no-shock conditioning paradigms. We did not find significant correlations between FOS activity and freezing behavior in any of the structures. However, this is not surprising given that freezing was highest in the paired groups, but FOS activity was highest in the BNST and BA in the unpaired groups. We used the non-parametric Spearman’s correlation analysis for each, as freezing behavior was not normally distributed. This is explained in the Methods (Statistical Analyses).

3. Why is there a difference in c-fos in central amygdala between the home cage and box-only groups?

Response: We initially analyzed home cage animals to obtain a measurement of baseline FOS activity. However, these are not the optimal controls, as they received different sensory input, and were not handled as frequently as the other behavioral groups. This could explain the increase in FOS in the CE in home cage animals compared to the no-shock group. We have removed these animals from the manuscript and re-analyzed our data. Figures 2-4 (and accompanying text) reflect these changes.

4. The 6-shock behavior data are not shown, and the design of the 3 versus 6-shock experiment is confusing. As described, it sounds like a new group of 5 female rats were trained post hoc and compared directly to the earlier groups of animals. This would of course be inappropriate- controls must be run in parallel.

Response: As described in the manuscript, there is evidence that females exhibit less context fear expression than males, although some find no sex differences. We included a group of female rats that received 6 shocks (run at the same time as the other behavioral groups) in an attempt to see whether stronger training could eliminate this freezing deficit. However, it is clear from the reviewer’s comment that this was a confusing addition to the manuscript and detracts from the overall paper. A thorough examination of context fear expression in females, the precise training conditions which lead to a deficit in learning and fear expression, and the accompanying changes in neural activation in the extended amygdala is needed, but beyond the scope of this paper. We have thus removed this experiment and Figure from the manuscript.

5. The c-fos quantification is not described adequately and it is impossible to tell how rigorously controlled the analysis was. The authors state that “two to three samples per hemisphere were counted and averaged together.” Two to three samples (which I assume means sections) would be a 50% difference between animals in the area sampled, and would mean that laterality was not counterbalanced or accounted for and that some animals had different regions analyzed. The numbers in the graphs are not normalized, and there is no indication of the area of tissue that was quantified or the variability in sampling area between animals. The example images do not appear to show the same AP level. An explanation of how the authors ensured that all animals had exactly the same regions and area sampled, and exactly where (AP/DV coordinates of the sample frame) should be included.

Response: We have expanded the description of FOS quantification (Methods: Microscopy) which addresses the issues raised by the reviewer, as the initial description was inadequate.

"For each brain analysis, we selected 80 micron slices covering the anterior-posterior axes of each structure (BNST: AP +0.12 - AP -0.36; CE: AP -2.50 - AP -3.0; BLA: AP -2.75 - AP -3.25). Delineation of the borders of each structure, and measurement of the area of each structure were made using NIS element software and ImageJ. ... FOS-positive cells were counted manually using ImageJ software. For each animal, we analyzed left and right hemispheres separately to reveal potential lateralization of FOS activity. Two to three samples per hemisphere were averaged together and divided by the surface area of the structure to normalize cell counts by area. Thus for each animal we obtained two measurements of FOS activity per brain structure: one measurement for the left hemisphere and one for the right hemisphere.”

The data in each bar graph are now normalized to the area of the structure. We have included Bregma levels in the lower right corner of each photomicrograph in each Figure.

6. The “random” tone-shock design is explicitly unpaired. The authors state that the “tone CS was designed to be a poor predictor of the shock,” but it is actually a negative predictor. The authors switch around the terms random, non-predictive, unpaired, unpredictable, and safety cue interchangeably, but these are not synonyms.

Response: We agree that the description of our experiment should be standardized throughout the manuscript: we now refer to this design as “unpaired”. Confusingly, previous studies have referred to this same design as “unpredictable” or “safety conditioning”. In the Discussion, we now make this explicit: that we use an unpaired training paradigm, and that this same paradigm has been used by others but referred to differently.

7. The activation of c-fos in basal amygdala in the unpaired groups is unsurprising, given that these groups experienced context fear conditioning and showed some generalization during testing. The surprising effect is the lack of c-fos activation in the LA of the males, and an explanation for this result should be offered.

Response: We now include this paragraph in the Discussion:

Surprisingly, expression of paired cued fear conditioning upregulated FOS in the LA in females but not in males. Many previous studies have demonstrated that the LA is required for the acquisition of cued fear conditioning and that acquisition upregulates the expression of immediate-early genes in the LA (Collins and Pare, 2000; Repa et al., 2001; Ploski et al., 2008). However, there are few studies measuring FOS in the LA following the expression of cued fear conditioning. Some find no increase in FOS in the LA (Hall et al., 2001); others find an increase in the most dorsal portion of the LA, but not in the ventral LA (Knapska and Maren, 2009), however these experiments were performed only in males. There is evidence that despite similar behavioral responses to cued fear conditioning, males and females might possess fundamental sex differences in fear circuitry structure (Gruene et al,. 2015). Thus, the possible differential contribution of the LA to fear expression in males and females should be explored further.
